# Improvement of steel alloys using indirect cooling grinding (I.C.G.)

**DOI:** 10.1016/j.heliyon.2023.e22738

**Published:** 2023-11-25

**Authors:** Ali Heydari, Masoud Pour, Mohammad Reza Gharib

**Affiliations:** a Department of Mechanical Engineering, University of Torbat Heydarieh, Torbat Heydarieh, Khorasan Razavi, Iran; b Department of Mechanical Engineering, Faculty of Advanced Technologies, Quchan University of Technology, Quchan, Iran

**Keywords:** Grinding, Surface roughness, Surface temperature, Indirect cooling, Depth of cut

## Abstract

Appropriate choice of machining conditions contributes directly to improved performance of the machining process. Cooling and lubricating the grinding surface in the machining process has been done using different methods, but each method has its own disadvantages. A new cooling system is proposed in this research to improve the surface roughness in flat-surface grinding. The workpiece is cooled using a mixture of water and antifreeze as a coolant, without directly contacting the cutting tool. The temperature of the workpiece surface remains fixed, and grinding of the workpiece is performed. This novel method has several benefits including no oxidation of workpiece and tool surfaces, no surface hardening from rapid cooling, no chip addition to the coolant, and extended grinding capabilities without replacement. The proposed methodology was tested on four steel alloys, including hot-worked and cold-worked steel, as well as two improved alloys. The tests involved changing various parameters such as the depth of cut, surface temperature, and coolant flow-rate, to analyze how they affected surface roughness. According to the results, the proposed method was remarkably efficient for low-chromium steel alloys. The best surface roughness was obtained using the indirect cooling system for the 1.1191 steel alloy (an improved steel alloy). In general, better results (lower roughness at higher cutting depth) were achieved at higher coolant flow-rates.

## Graphical abstract

**Figure:**
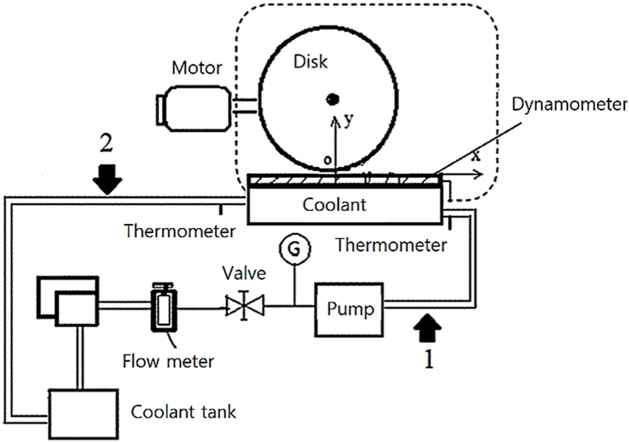

## 1 Introduction

Manufacturing processes have always aimed to achieve higher production rates and produce high-quality parts. Machining covers a range of material removal procedures, including but not limited to turning, milling, and grinding, which are considered as fundamental methods within this basic group. Many kinds of research and studies have been performed and investigated the machining parameters [[1], [2], [3]]; surface roughness [[4], [5]], and coatings [[6], [7]]. But the grinding has been the main finishing process for achieving the aforementioned objectives. More than 42 % and 25 % of all machining tools and machining processes in the United States are related to grinding, respectively [8]. With the advancement of grinding machine technology, the economic efficiency of these machines has improved significantly. Thus, the material removal rate and grinding production rate have rapidly approached those of the turning and milling processes.

Numerous investigations have been performed to understand the grinding process better and improve its performance; these works have focused on six main parameters: surface roughness, temperature, force, grinding wheel topography, chattering vibration, and wear. Among others, surface roughness has always been the ultimate factor to be controlled. Although thermal analysis of grinding is complicated, this field of research has been improved due to the studies by [9], who proposed a comprehensive thermal model of grinding that has been widely used in grinding problems since then. His study was later on republished in the form of a book [10].

[11] examined various aspects of grinding in a series of eight articles titled "Applied Mechanics in Grinding." They analyzed the workpiece's residual stress and metallurgical structure using Malkin's thermal model and further elaborated on the effects of the coolant on the workpiece structure. Recently, many researchers have focused on the role of the grinding environment in surface quality and grinding forces. ([[12], [13]], investigated the effect of using liquid nitrogen as a coolant to improve the grinding forces and surface roughness in a cryogenic grinding process. They tested 316 stainless steel and AISI D3 in dry, humid, and cryogenic environments and showed that decreasing the grinding temperature would improve the machining performance. They also reported higher grinding efficiencies at higher liquid nitrogen pressures.

[14] focused on grinding low-carbon steel with coated wheels under dry, cryogenic, and lubricated conditions, highlighting the superiority of the lubricated rather than the cryogenic condition. Devising a carbon nano-lubrication system [15], improved the surface quality of aluminum alloy (AL-2017-T4, used in the aviation industry) at lower grinding forces. They showed that the use of carbon nano-lubrication, rather than conventional lubrication, increased the shear force and surface roughness by 21.99 % and 46.32 %, respectively. Other researchers have investigated using different nanoparticles for grinding different alloys [[16], [17], [18]].

[19] investigated the effects of temperature and energy on the grinding process with minimum quantity lubrication (M.Q.L.), concluding that the contributions of energy and local temperature were affected by the abrasive material and surface cooling/lubrication conditions [20]. reported a temperature analysis on M.Q.L. grinding, where they investigated the effects of oil pressure, oil volumetric flow-rate, and characteristics of the oil droplet on the grinding process. They also explored the limitations of this method for the AISI 4140 steel alloy [21]. In another study [22], examined the effect of liquid spraying on the grinding process of aluminum oxide ceramics through an M.Q.L. procedure. The oil spraying conditions, including the nozzle angle and distance, were studied and optimized. [23] comprehensively overview the M.Q.L. method for all machining processes, including grinding.

[24] experimentally investigated the grooved stone grinding process with coolant spraying. They showed that the grooves on the grinding wheel increased the rate of heat transfer through the droplets and hence accelerated the cooling of the workpiece, thereby lowering the energy consumption for cooling [25]. considered the cooling performance of the M.Q.L. grinding process with different vegetable-based oils as nanofluid coolants, suggesting the superiority of low-viscosity high-surface tension vegetable-based oils for cooling and the high-viscosity, high-surface tension vegetable-based oils for lubrication purposes. In another study [26], proposed an enhanced cooling system for the grooved grinding wheel [27]. reviewed the various cooling methodologies used in machining processes, including cryogenic cooling, minimum-quantity lubrication, high-pressure coolants, solid lubricants, flood cooling, and dry machining.

In experimental work, [28] compared the produced surface roughness and hardness of MO40 steel upon subjecting the steel to a grinding process with different cooling techniques. Their results indicated that for lower depths of cut and cutting velocities, better surface quality could be achieved with liquid nitrogen or CO_2_ rather than a lubricating fluid; for higher depths of cut and cutting velocities, however, the produced surface hardness was higher with either liquid nitrogen or CO_2_ gas rather than lubricating fluid [29]. analyzed the machined surface of SAE 4340 steel upon grinding with different cooling-lubrication techniques, such as M.Q.L. and M.Q.L. with a cleaning system (M.Q.L. + C.S.). According to the results, larger bearing areas and lower peaks and valleys dispersion were observed on the surfaces that were ground with the M.Q.L. and M.Q.L. + C.S. systems. In another experimental work [30], evaluated an eco-friendly MQL-assisted grinding process that was cooled by a graphene-enhanced plant-oil-based cutting fluid (POBCF), where the graphene nanoparticles (GPNPs) were added to the POBCF to improve the grinding components of the TC4 alloy. The research results highlighted the positive effects of the GPNP on the grinding characteristics [31]. evaluated surface integrity in grinding of Inconel 718 superalloy by employing silver and zinc oxide (ZnO)-based ecological nanofluids in a minimum quantity lubrication (M.Q.L) mode. Their finding suggests that ZnO-based nanofluid have superiority over the silver-based nanofluid using M.Q.L mode in terms of reduced grinding forces and favorable residual stress. For this alloy [32], experimentally investigated the tribological performance of Nanofluid Minimum Quantity Lubrication (NF.M.Q.L) technique using vegetable oil as the base oil. They show that vegetable oil based NF.M.Q.L technique enhanced the grinding performance of Inconel 718 alloy in terms of surface roughness, grinding energy and G-ratio because of a thin stable lubrication film formation at the tool-workpiece interface [33]. theoretically and experimentally studied the feasibility of M.Q.L assisted belt grinding of titanium alloys. As their results, the addition of carbon nanotubes in grinding fluids was confirmed to be an effective way to further improvement in the effect of M.Q.L.

Given the paramount importance of the cooling system in the resultant surface roughness of the workpiece upon a grinding process, the present research aims at investigating the parameters affecting the cooling function and strategies for optimizing this function. The direct use of coolant during the grinding process is associated with particular challenges such as oxidation of the workpiece surface, surface hardening due to rapid cooling, and high consumption rate and limited recyclability of the shear and coolant fluid (lubricants). Hence, we propose an innovative Indirect Cooling Grinding (I.G.C.) system where the workpiece is not in direct contact with the coolant. Here the coolant is not sprayed at the workpiece-grinding wheel interface (as is practiced in conventional methods) but is rather in contact with the lower surface of the workpiece only to cool it down to the desired temperature. Since no chip is introduced into the coolant and the coolant undergoes no direct contact with the shear area, it can be used for extended working hours without replacement. As the research gap in the field of grinding process, no research in the field of dry grinding has investigated the indirect cooling of the surface from the bottom of the workpiece. Regarding the shift in the grinding industry to M.Q.L., semi-dry, and dry methods, the proposed method offers a large potential for achieving optimal grinding performance. Some advantages of this method are:

• No oxidation of the workpiece and tool surfaces
• No surface hardening due to rapid cooling
• No chip is introduced into the coolant (recyclability of the coolant)
• Extendable for more working hours without replacement

Thus, this method is applicable and suitable for long machining without the need to replace the workpiece and recycle the coolant. This experimental research aims to evaluate the surface roughness of four classes of steel alloys upon implementing the proposed cooling system (I.C.G.). Accordingly, the surface roughness was measured and compared at different flow-rates, coolant temperatures, and cutting depths. The results indicated the suitability of the proposed method for improving the surface quality of the studied steel alloys.

## 2 Method and material

### 2.1 Indirect cooling grinding (I.C.G.)

Considering heat-induced damage as one of the significant limitations of the grinding process, the cooling system plays an essential role in this process. Cooling and lubrication play essential roles in grinding to avoid heat-induced damage to the workpiece surface due to high friction and severe heat generation. The I.C.G. is herein proposed as an innovative method for cooling steel parts during the grinding process. Here the coolant, which is a mixture of water and ethylene glycol-based antifreeze, is cooled down to the desired temperature using an indirect cooling system. Subsequently, the workpiece temperature is maintained throughout the grinding process by adjusting the volumetric flow-rate of the coolant. The proposed cooling system was evaluated by measuring the produced surface roughness using a contact-type roughness instrument upon applying the I.C.G. on four steel alloys at different cutting temperatures and depths.

### 2.2 Flat grinding machine

The grinding operation was carried out by a flat grinding machine (WNX 32.11, made in Romania). The machine table was driven by hydraulic force, and the wheel force was measured by a micrometer within ±0.001 mm. The grinding machine was equipped with a white aluminum oxide wheel (manufactured by Olimeta Company, standard designation: WA46K). The machine specifications are tabulated in Table 1. All tests were performed with constant grinding wheel diameter and rotation speed. Table 1 provides complete information on rotation speed and grinding wheel diameter. The selected depth of cut range is usually used in the early stages of milling and the effects of these machining conditions can change the surface roughness and surface hardness of the workpiece and make it difficult to achieve the desired surface roughness and hardness in the final workpiece. Therefore, the machining conditions are selected in such a way that firstly generate significant heat and, secondly have a noticeable effect on the surface roughness and hardness of the workpiece surface.

**Table 1:** Nominal specifications of the grinding machine model WNX 32.11

Specification	Amount
Plate area	500mm × 200 mm
Outer Diameter of spindle	250 mm
Inner Diameter of spindle	76 mm
Spindle speed	280RPM
Feed and Cross-Feed	0.08 m/s

### 2.3 Cooling system

**Fig. 1:**
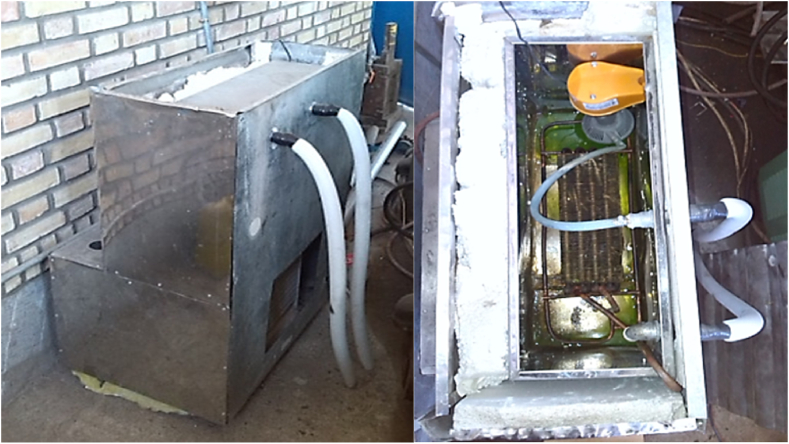
The view of the cooling device.

The cooling was provided by a vapor compression refrigeration cycle (VCRC) in the experimental tests. A vapor compression refrigeration cycle (VCRC) is applied as the cooling system because of cost-effective and easily implementation. The cycle was enhanced by applying modifications to the cooling water system by adjusting the system height to establish the natural return of the flow from the workpiece box, boosting the evaporator, and installing a water pump in the tank. The coolant used in this experiment was a mixture of water and ethanol-based antifreeze (to minimize the environmental degradation and corrosive effects on metals) at a blending ratio of 1:1, which could withstand freezing at temperatures down to −40 °C. Fig. 1 demonstrates the cooling system.

### 2.4 Workpiece box

**Fig. 2:**
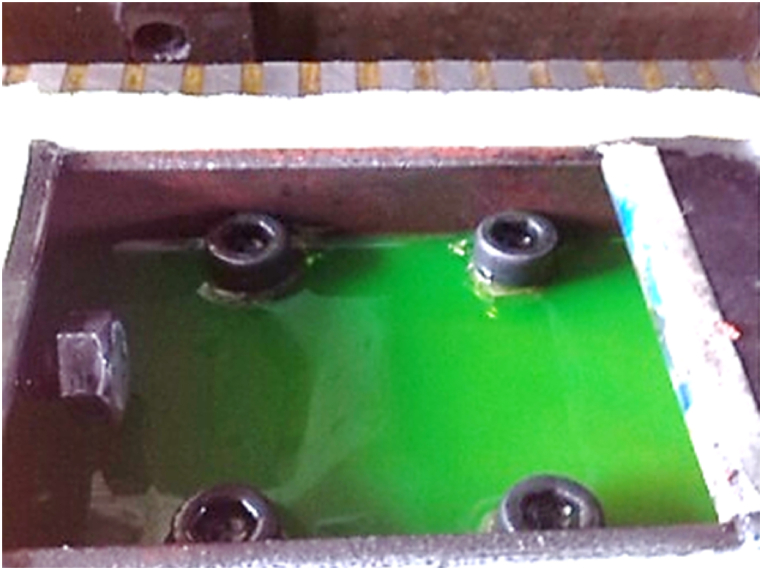
The view of the special box for holding the workpiece.

The workpiece box was designed, so that an embedded screw fixed the workpiece, and the workpiece height could be adjusted by four Allen bolts (Fig. 2). The box dimensions were 80 × 200 × 50 mm, with a coolant capacity of 0.8 L. This box is the workpiece immersion container that contains the coolant around the workpiece. It forms a thin coolant film on the upper surface to remove the chips. Therefore, we now perform both workpiece immersion and grinding surface cleaning instead of just spraying coolant.

**Fig. 3:**
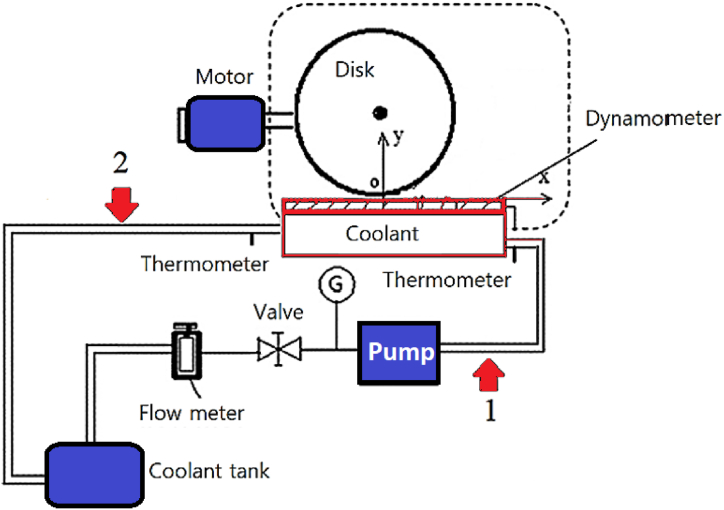
A schematic of the whole system.

Two input and outlet valves have been placed to allow for manual control of the volumetric flow-rate of the coolant. The coolant fluid was used to partly submerge the workpiece, with the grinding surface left exposed. The flow-rates of the coolant were set at 15 and 40 mL/s, respectively. The box was made from iron to hold a grinding machine on top of it by establishing a magnetic force. It was also insulated to prevent energy loss. Two high-sensitivity k-type temperature sensors were installed to measure inlet and outlet temperatures. A schematic view of the whole system is shown in Fig. 3. This figure shows that in path 1, the coolant comes out of the coolant tank and is adjusted by a flow regulation sensor located near the pump. Then the coolant inlet and outlet of the workpiece box were measured using two thermometers, and the coolant in path 2 was returned to the coolant tank. The shear forces *F*_*n*_ and *F*_*t*_ were measured using a Kistler 9255B dynamometer.

### 2.5 Measurement equipment and used material

Two k-type temperature sensors were used to measure the coolant temperature at the inlet and outlet of the workpiece box. Additionally, a laser thermometer was used to measure the surface temperature of the workpiece during the grinding process. In order to calibrate the laser thermometer for steel, an emission coefficient of 0.8 was used according to the machine instructions. After each test, the surface roughness of the workpiece was measured by a PCE-RT2200 roughness tester (made in Germany) - a contact-type device with a displacement sensor of 2.4 mm.

Basic concepts, such as cut-off length and base length, were used to measure the surface roughness. The distance traveled by the needle is called the cut-off length, which is generally in the range of 0.08–25 mm. Many engineering applications opt for a cut-off length of 0.8 mm. On the other hand, the cut-off length should be large enough to measure 10 to 15 roughness irregularities in addition to the surface wave. In all the measurements performed in this work, the cut-off and base lengths were set to 0.8 and 4 mm, respectively. The grinding experiments were performed on four alloy groups as follows:

(A) hot-rolled steel group suitable for plastic molding (defined in DIN standard as 1.2312)
(B) hot-rolled steel group suitable for metal injection (defined in DIN standard as 1.2344).
(C) Cold-work tool steel group (defined in the standard as DIN 1.2080)
(D) Improved steel group (defined in DIN standard as 1.1119).

The properties and elemental compositions of these alloys are listed in Table 2 [34] ((DIN)).

**Table 2:** The properties and percentages of the elements present in the above alloys.

Element	Alloy 1.2312	Alloy 1.2344	Alloy 1.2080	Alloy 1.1119
**Carbon**	0.4	0.39	1.9 to 2.2	0.42 to 0.5
**Chrome**	1.9	5.1	11 to 12	0
**Molybdenum**	0.2	1.3	0	0
**Phosphorus**	0.035	0.035	0.03	0.035
**Sulfur**	0.035	0.035	0.03	0.035
**Manganese**	1.5	0.4	0.15 to 0.45	0.5 to 0.8
**Silicon**	0.4	1.1	0.1 to 0.4	Less than 0.4

### 2.6 Experiments

Based on the discussion presented in Section 2, the grinding factors (*i.e.,* depth of cut, advancement of grinding wheel, and table feed rate) were set as detailed in Table 3. It is worth mentioning that preliminary experiments were performed at cutting depths of 0.001, 0.002, 0.003, 0.01, 0.02 and 0.03 mm, followed by omitting the cutting depths of 0.001, 0.002 and 0.003 mm because of the insignificant heat generation. The working grinding wheel spindle speed (R.P.M.) and the table's feeds (in two directions, including feed and cross-feed) were kept constant. Table 3 further states the values of the coolant temperature and volumetric flow-rate. The dimensions of the workpiece were designed so that the workpiece box could be easily deployed and the grinding wheel could grind the entire workpiece surface in one step.

**Table 3:** The values of variable and fix parameters of the problems.

parameters	values
cutting depths (mm)	0.05, 0.1, 0.15, 0.2, 0.25
Feed (m/s)	0.08
Temperature (°c)	0, −10
volumetric flow rate (cc/s)	0, 15, 40
Cross-wheel drive (mm)	16

**Fig. 4:**
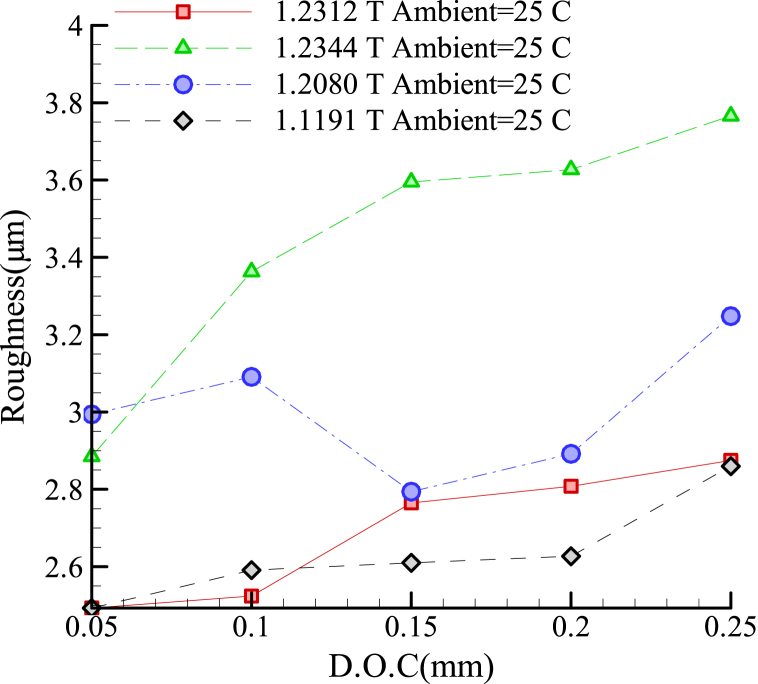
Roughness in various alloys under grinding without cooling.

The workpiece dimensions were 80 × 80 mm; it was manufactured by vertical milling with a uniform and controlled surface roughness of 6 μm. In all experiments, the surface roughness was produced with only one pass of the grinding wheel. The surface roughness of samples is measured by the conventional Ra parameter in industrial workpieces. The roughness measurement device was portable. To avoid operator-related errors, a reference point at the center of each workpiece was considered, and the surface roughness measurement was performed at this point in triplicates. Finally, the mean was recorded as the final surface roughness. In order to attain a better understanding of the effectiveness of the proposed I.C.G. method, several experiments were performed with no coolant. Fig. 4 is the basis for calculating the percentage of roughness improvement of I.C.G. Processes. As seen in this figure, the surface roughness increased with the cutting depth regardless of the alloy type. However, the increase was more pronounced for the 1.2344 steel alloy.

The changes in the surface roughness upon deploying the I.C.G. method for each of the alloys are discussed in the following. Based on the results, the 1.1119 alloy exhibited superior surface roughness upon the grinding process, while the 1.2344 alloy achieved the highest roughness values.

## 3 Results

### 3.1 Alloy #1: 1.2312 steel

**Fig. 5:**
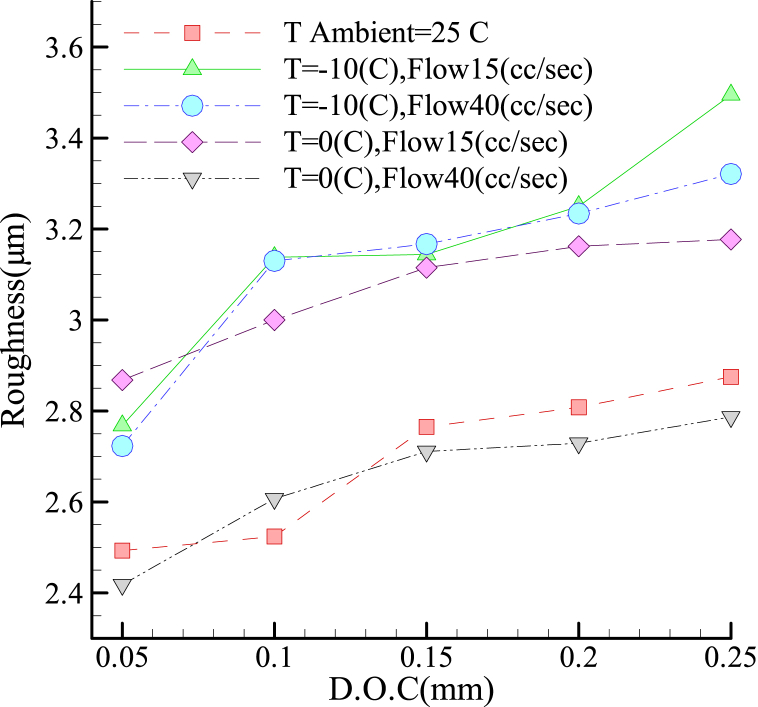
Roughness in terms of cutting depth for grinding alloy 1.2312 with the I.C.G. method.

As shown in Fig. 5, for the 1.2312 steel alloy, the increase in the cooling fluid volumetric flow-rate at −10 °C imposed no considerable effect on the roughness. However, at 0 °C, any increase in the coolant volumetric flow-rate improved the surface quality and reduced the roughness. The best conditions in roughness were obtained with I.C.G. at a coolant volumetric flow-rate of 40 mL/s and temperature of T = 0 °C. Furthermore, it was observed that increased cutting depths resulted in greater roughness values, although the upward trend was more conspicuous at lower temperatures. Overall, the implementation of the I.C.G. technique resulted in improved surface roughness when compared to the condition without the coolant, but only at extremely low workpiece temperatures (approximately zero) and high volumetric flow rates of the coolant. At lower temperatures, the cooling rate of the workpiece surface is higher. Therefore, the temperature reduction rate for the enhanced surface increases, as shown in Fig. 13, in this situation the harder structures is formed. To examine the surface microstructure, an SEM image of the surface microstructure at a temperature of −10 °C has been provided.

### 3.2 Alloy #2: 1.2344 steel

**Fig. 6:**
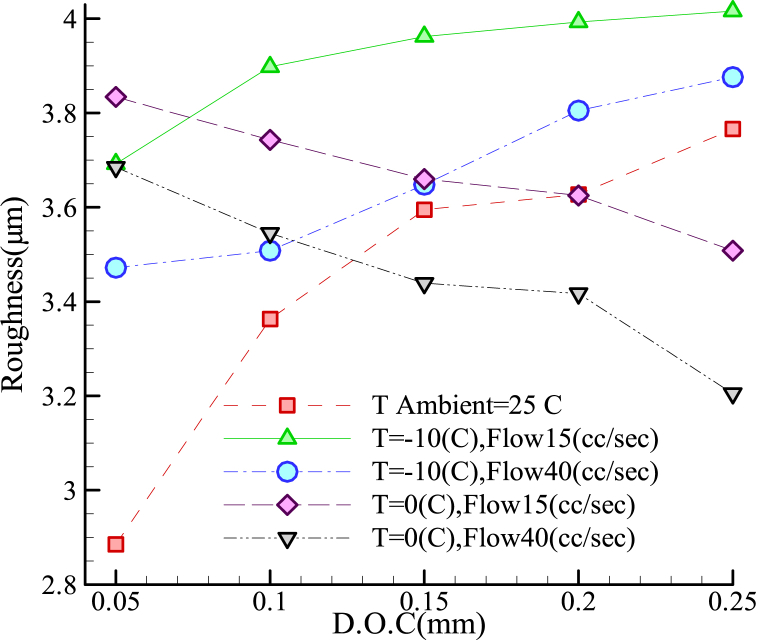
Roughness in terms of cutting depth for grinding alloy 1.2344 with the I.C.G. method.

As shown in Fig. 6, for the 1.2344 steel alloy, at 0 °C, the surface roughness was improved by increasing the volumetric flow-rate from 15 mL/s to 40 mL/s. In the given circumstances, irrespective of the coolant temperature (−10 °C or 0 °C), raising the volumetric flow rate of the coolant resulted in a significant reduction in surface irregularity. Moreover, lower surface roughness values were obtained at lower cutting depths when the coolant was absent. The patterns of surface roughness variations were different for the two tested flow-rates (15 and 40 mL/s). At −10 °C, the surface roughness increased with the cutting depth, while it was improved with increasing the cutting depth at 0 °C.

The best conditions for surface roughness included a high cutting depth, a temperature of 0 °C, and a flow-rate of 40 mL/s. The cutting depth exhibited a dual effect; in this respect, an increase in the cutting depth at 0 °C reduced the surface roughness, while the same increase in the cutting depth added to the surface roughness in the absence of coolant at −10 °C. Thus, based on Fig. 6, rapid cooling of the 1.2344 steel alloy at a coolant temperature of −10 °C was not favorable. As can be seen in Fig. 5, Fig. 6, for the 1.2312 and 1.2344 steel alloys, improved workpiece surface roughness was obtained at higher cutting depths by applying the zero-Celsius coolant at 40 mL/s.

### 3.3 Alloy #3: 1.2080 steel

**Fig. 7:**
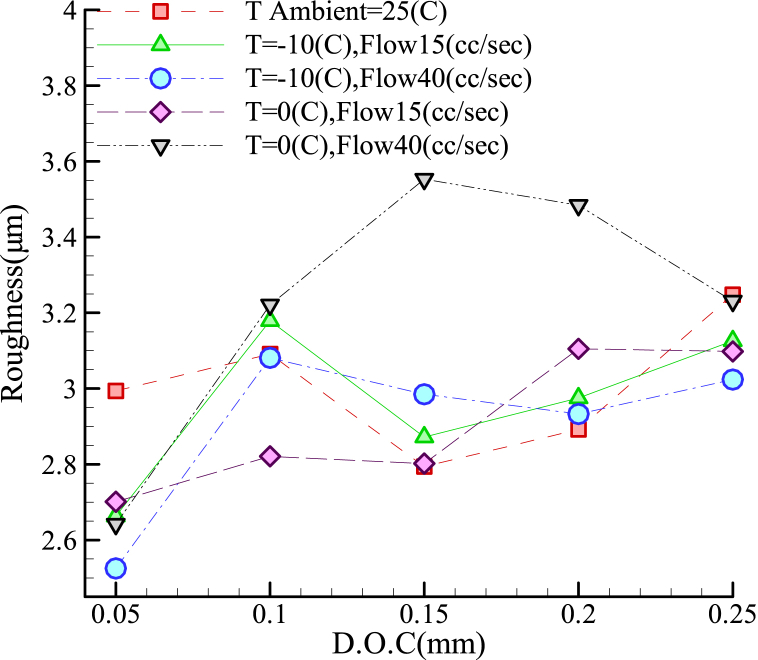
Roughness in terms of cutting depth for grinding alloy 1.2080 with I.C.G. method.

As shown in Fig. 7, focusing on the 1.2080 steel alloy, the use of the coolant led to fast surface hardening. In this respect, unlike in the previous case, the application of the coolant at a volumetric flow-rate of 40 mL/s at 0 °C increased the workpiece surface roughness. Fig. 7 further shows the optimality of flow-rate of 15 mL/s at 0 °C for cutting depths below 0.1 mm. At other cutting depths, no significant difference was observed between the I.C.G. and the coolant-free grinding because of the nonlinearity of the material behavior. This might be attributed to the alloy's chromium content (11–12 %). Although the alloy did not exhibit a specific trend of variation, the best performance occurred at a cutting depth of 0.05 mm, a volumetric flow-rate of 40 mL/s, and a coolant temperature of −10 C.

### 3.4 Alloy #4: 1.1191 steel

**Fig. 8:**
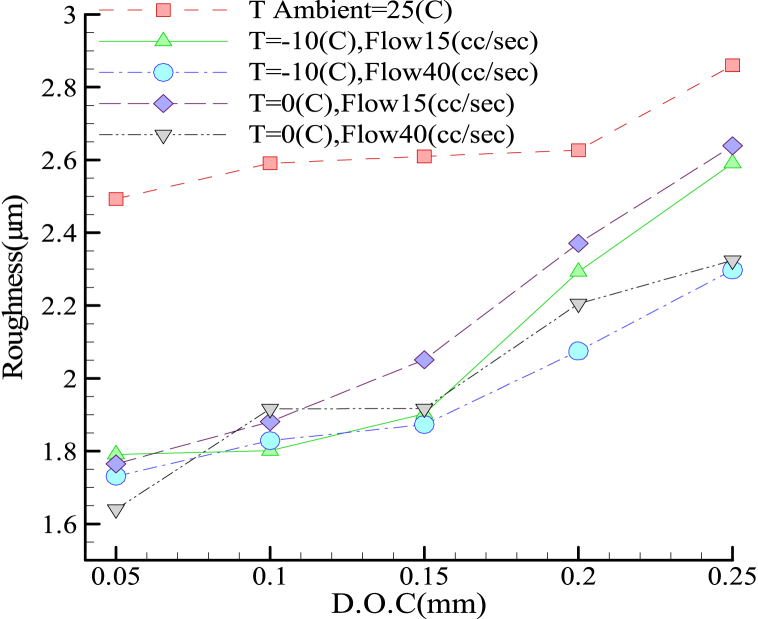
Roughness in terms of cutting depth for grinding alloy 1.1191 with I.C.G. method.

Fig. 8 shows the plot of surface roughness versus cutting depth for the grinding process of the 1.1191 steel alloy by the I.C.G. method. With zero chromium content, this alloy was supposed to be free of heat-induced material hardening and improve its surface roughness by increasing the coolant volumetric flow-rate and/or decreasing the temperature. Optimal results were obtained at a coolant flow-rate and temperature of 40 mL/s and −10 °C, respectively, while the worst were observed in the absence of neither the coolant nor the I.C.G. system.

**Fig. 9:**
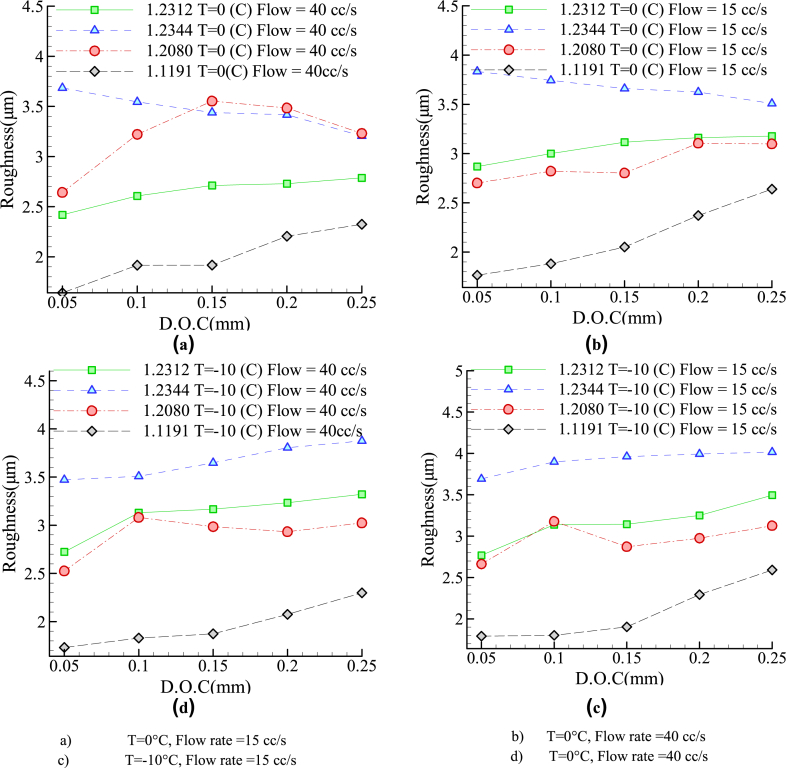
The roughness of different alloys at different temperatures and volumetric flow rates under I.C.G. grinding conditions a) T = 0 °C, Flow rate = 15 cc/s b) T = 0 °C, Flow rate = 40 cc/s c) T = −10 °C, Flow rate = 15 cc/s d) T = 0 °C, Flow rate = 40 cc/s.

On the other hand, at lower cutting depths, any change in coolant temperature and/or volumetric flow-rate altered the surface roughness steadily, while improved surface roughness was achieved at higher cutting depths. Fig. 9 compares different steel alloys (in terms of surface roughness) with different coolant temperatures and volumetric flow-rates under the same I.C.G. system. According to this figure (Fig.s 9 (a), 9(b), 9(c) and 9(d)) irrespective of the coolant temperature and flow-rate, the best and the worst conditions were related to the 1.1191 and 1.2344 steel alloys, respectively. According to Fig.s 9 (a) to 9 (d), For alloy 1.2344, The process of changes in surface roughness is different according to the depth of cut at 0 °C and −10 °C.

Table 4 indicates the cooling conditions for achieving better roughness for each alloy compared with grinding without cooling. It must be explained that other cooling conditions not listed in this table do not improve surface roughness. According to this table, it can be seen that for steel 1.1911 for all cooling conditions, the roughness is improved in comparison with grinding without cooling. The cooling condition for the best roughness improvement for 1.2312 and 1.2344 alloys is 0 °C temperature and 40 cc/s flow rate for 0.25 mm depth of cut. For 1.2080 alloy the best roughness improvement is related to −10 °C temperature and 40 cc/s flow rate for 0.05 mm depth of cut. Finally, 34.22 % improvement in roughness is achieved by 0 °C temperature and 40 cc/s flow rate for 0.05 mm depth of cut for 1.1911 steel alloy.

**Table 4:** List of the cooling conditions for achieving better roughness for each alloy in comparison with grinding without cooling.

Steel Alloy	Flow rate (cc/s)	Temp. (°C)	D.O.C (mm)	Percentage of Roughness improvement (%)
1.2312	40	0	0.15	1.8
			0.2	2.7
			0.25	**3.1**
1.2344	40	0	0.15	4
			0.2	5.7
			0.25	**15**
	15	0	0.25	6.9
1.2080	40	−10	0.05	**15.67**
			0.25	6.9
		0	0.05	11.75
	15	−10	0.05	11
			0.25	3.7
		0	0.05	9.8
			0.1	8.7
			0.25	4.6
1.1191	40	−10	0.05	30.56
			0.1	29.4
			0.15	28.23
			0.2	21
			0.25	19.65
		0	0.05	**34.22**
			0.1	26
			0.15	26.55
			0.2	16
			0.25	18.74
	15	−10	0.05	28.16
			0.1	30.5
			0.15	27.1
			0.2	12.7
			0.25	9.41
		0	0.05	29.2
			0.1	27.4
			0.15	21.4
			0.2	9.74
			0.25	7.73

## 4 Discussion

### 4.1 Investigation of metallography structure of workpieces

**Fig. 10:**
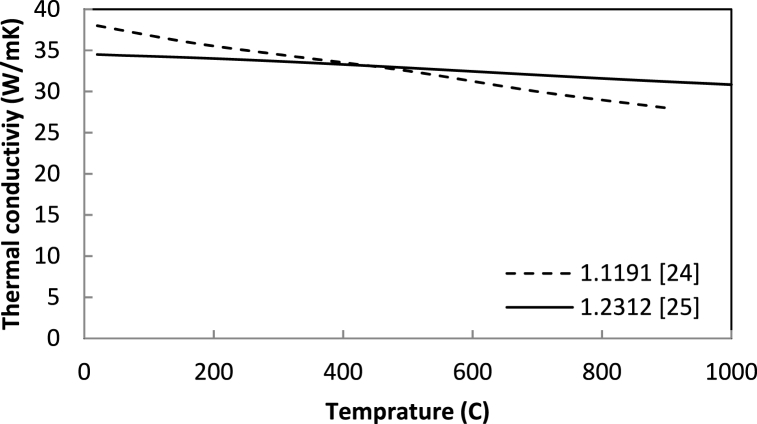
The comparison of thermal conductivity of 1.1191 with 1.2312.

The cooling path of the workpiece during cooling consists of several phases and the percentage of stabilized phases in the workpiece changes based on the cooling rate. The temperature of AISI 1.1191 and AISI 1.2312 steels are approximately equal to 900 °C before beginning of the cooling process. Therefore, their cooling rate at different flow and coolant temperatures is almost identical. The following figure (Fig. 10) compares the heat transfer coefficient of two steels [[35], [36]]. Thus, it can be expected that the cooling path of the steel will be similar to the C.C.T. diagram. The cooling path of steels is measured using non-contact laser measurements by determining their surface temperature.

The C.C.T. diagram contains curves in the linear coordinates of temperature and logarithm of time. It determines the beginning and end of the austenite phase conversion to other phases for each cooling path. The process of cooling the steel and passing through each phase confirms the formation of different phases in the steel structure.

The formation of hard phases upon cooling is a major factor contributing to the ultimately generated. As stated in Ref. [37], the cooling rate of steel can affect its structural phases. On the other hand, it has been shown in Ref. [38] that the steel phases vary at different depths relative to the surface, depending on the temperature distribution created across the steel and surface cooling operations. The temperature distribution can change the steel structure down to a depth of about 1 mm from the surface [38]. On the other hand, the temperature distribution established upon cooling the workpiece via its upper surface differs from that via its lower surface, depending on the thermal conductivity of the alloy.

**Fig. 11:**
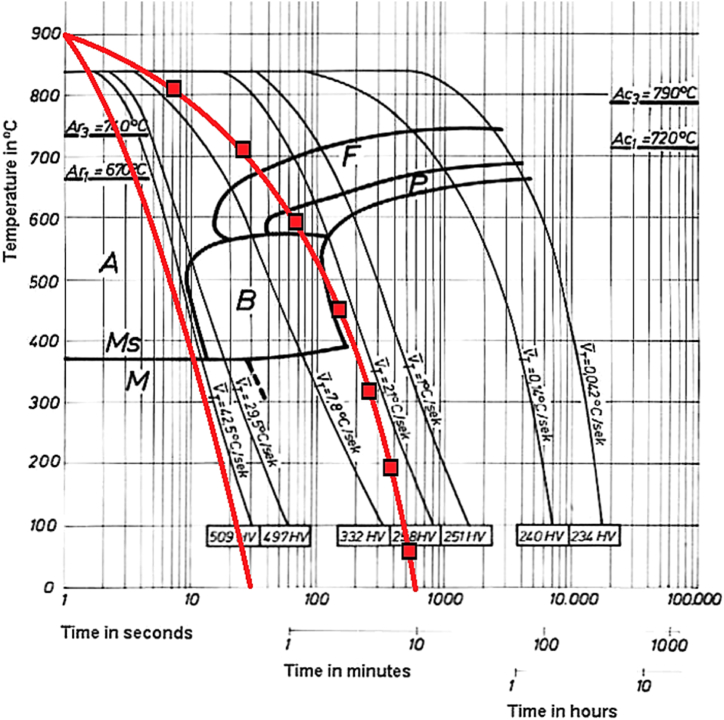
C.C.T. diagram of 1.1191 [40] and the selected cooling direction by coolant flow A) (▬) 40 cc/s and −10 °C B) line with Square sign(▬■▬) 15 cc/s and −10 °C.

According to finite element simulations performed for the grinding process, previous research shows that the temperature increases to about 980 °C on the workpiece surface [39]. However, the short heating time and the presence of the coolant act to cool down the workpiece rapidly. Respecting the very short workpiece cooling time, the phase transformation in the steel should be examined based on the continuous cooling transformation (C.C.T.) diagrams. To investigate the effects of the coolant temperature and flow rate on the steel structure, two opposite states developed in the 1.2312 and 1.1191 steel alloys have been examined. According to Ref. [37], for the 1.1191 steel alloy, should the pearlite phase exists, a higher cooling rate will lead to a greater pearlite-ferrite phase, and the bainite phase will be formed eventually (Fig. 11). Given the cooling rate of the 1.1191 steel alloy, the bainite phase formation was expected at −10 °C and 40 mL/s. This is evident in the C.C.T. diagram for the 1.1191 steel alloy (Fig. 11), where the bainite phase is actually formed at −10 °C for coolant flow rates of 40 mL/s. As shown in Fig. 11, the cooling path is cooled to −10 °C, and a flow rate of 40 mL/s passes through the bainite phase range and causes this phase to form in the steel. But by slowing down the cooling speed and creating the path ▬■▬ the pearlite and ferrite phases can be formed. Due to the small volume of hard phase formed, no noticeable change in surface roughness has occurred.

**Fig. 12:**
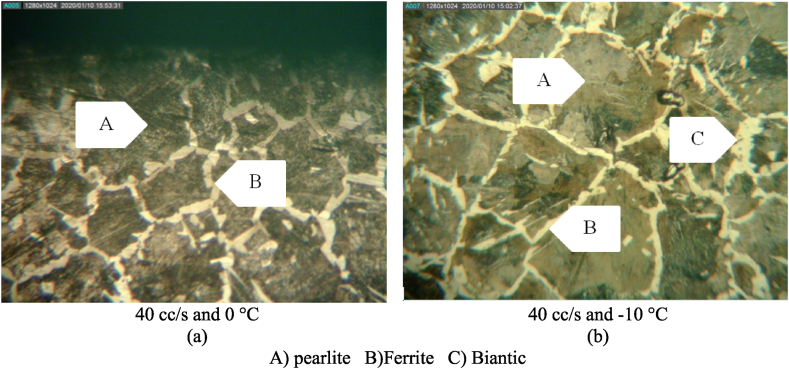
Microstructure of Steel 1.1191 in different machining conditions a) at flow rate of 40 cc/s and temperature of 0 °C b) at flow rate of 40 cc/s and temperature of −10 °C.

Due to the hardness of the bianitic phase, its volume can affect the hardness and roughness of the resultant surface. The hard phase boosts the wheel oscillations by increasing the force applied to the grinding wheel, hence the surface roughness. Fig. 12 shows that the formation of the bainite phase was limited, leading to improve surface roughness values due to the size of the ferrite structure. This was the main reason behind the decrease in the surface roughness at coolant flow-rates of 40 and 15 mL/s at −10 °C, as compared to those at 0 °C (Fig. 8). Thus, as stated by Ref. [41], with the increased cooling rate in steel 1.1191, the possibility of martensite and bainite phases has increased.

**Fig. 13:**
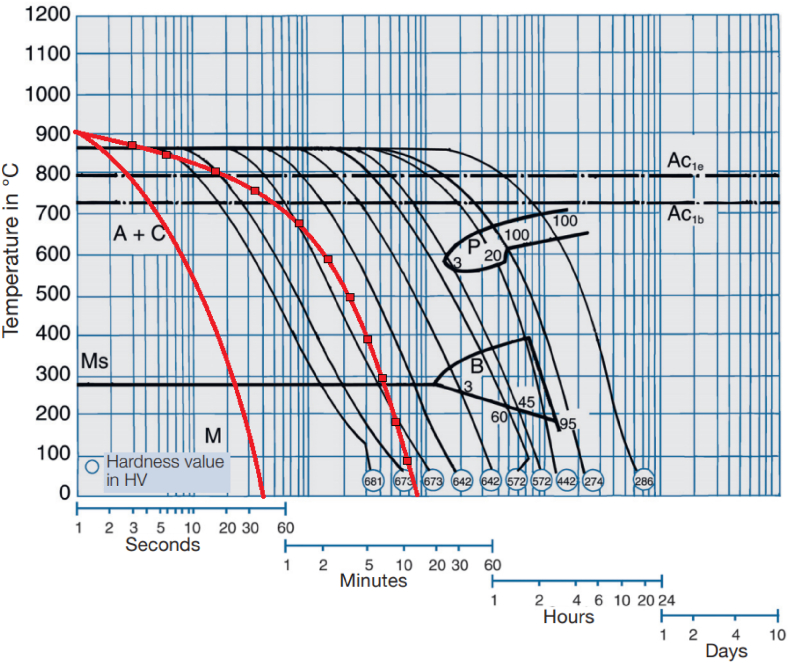
C.C.T. diagram of 1.2312 [40] and the selected cooling direction by coolant flow A) (▬) 40 cc/s and −10 °C B) (▬■▬) 15 cc/s and −10 °C.

On the other hand, as expected, the C.C.T. diagram (based on the cooling data in this research – the solid blue line) drawn in Fig. 13 depicts that the high cooling rate of the workpiece at coolant flow-rates of 40 and 15 mL/s and coolant temperature −10 °C led to the formation of a large volume of martensitic phase for the 1.2312 steel alloy. As expected, with decreasing coolant temperature, the martensite phase formation in 1.2312 steel increased sharply. This can be understood by comparing paths (▬) and (▬■▬).

**Fig. 14:**
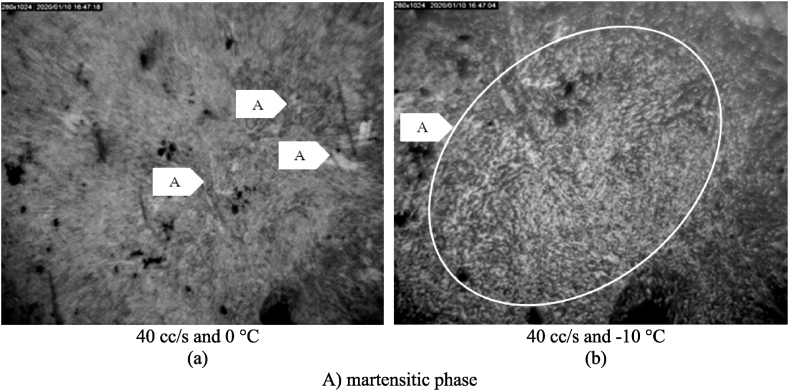
Microstructure of Steel 1.2312 in different machining conditions a) at flow rate of 40 cc/s and temperature of 0 °C b) at flow rate of 40 cc/s and temperature of −10 °C.

The increase in the volume of the martensitic phase could enhance the surface roughness by improving the hardness of the steel at the time of cutting. To check for this effect, the surface texture of the machined workpieces of 1.2312 steel alloy at a coolant flow-rate of 40 mL/s and coolant temperatures of −10 °C and 0 °C are shown in Fig. 14. The figure shows that an increase in the martensitic phase volume adds to the surface roughness, confirming the results presented in Fig. 5.

**Fig. 15:**
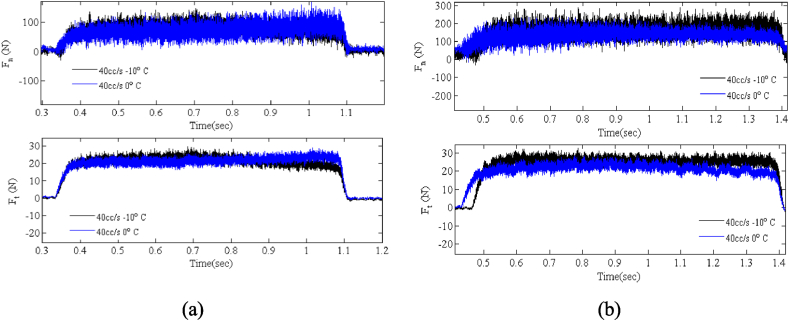
The changes of grinding forces (a) steel 1.1191 (b) steel 1.2312.

A comparison between the test conditions in Fig. 12, Fig. 14 has been performed to evaluate the changes in grinding forces in different grinding conditions. As shown in Fig. 15 (a), in steel 1.1191, the variations made in the phases do not cause much change between the machining forces. But in steel 1.2312 (Fig. 15 (b)), changes can be seen in two modes of machining conditions. So, vertical forces do not change with decreasing temperature, but tangential forces increase slightly. Note that the changes are due to the formation of martensite phases.

Because the surface of the workpiece receives the most heat in contact with the wheel, the heat treatment generated in the workpiece varies based on the temperature variation at different depths of the workpiece. Thus, heat treatment is performed on the surface in the I.C.G. method. The purpose is to determine the surface structure based on the depth of the workpiece in order to determine the effectiveness of this cooling method in changing the phases of the workpiece. In other words, the authors have considered the creation of surface roughness to be related to the non-removal of hard materials created during machining. This issue has also been confirmed by examining the machining forces. There is an increase in machining force in the case of the formation of hard phases (such as 40 cc/s flow rate and −10 °C temperature in the workpiece for steel alloy 1.2312). This is also confirmed by the simultaneous examination of the surface texture in Fig. 14.

### 4.2 Selection of the optimal conditions

This section examines only the most desirable condition among the existing conditions to achieve a more appropriate roughness and hardness.

#### 4.2.1 Optimal conditions for economic efficiency

The number of cutting depths is a crucial factor contributing to the time taken to achieve the final dimension of the workpiece. Hence, the designer may seek to realize a higher cutting depth in terms of economic efficiency. According to the presented diagrams, the use of I.C.G. with the coolant at 40 mL/s and 0 °C led to optimal results in terms of economic efficiency in the 1.2312 steel alloy. The corresponding optimal conditions for the 1.2344, 1.2080, and 1.1191 steel alloys were 40 mL/s and 0 °C, 40 mL/s and −10 °C, and 40 mL/s and −10 °C, respectively. Thus, the better results for economic efficiency (lower roughness at higher cutting depth) were achieved at higher coolant flow-rates. In grinding process, low depth of cut can lead to increasing temperature and surface roughness due to several reasons.

• Firstly, when the depth of cut is low, the contact area between the grinding wheel and the workpiece is small. This results in a higher specific energy input, which generates more heat in a smaller area. As a result, the temperature of the grinding zone increases.
• Secondly, low depth of cut can cause the grinding wheel to rub against the workpiece instead of cutting it. Rubbing generates more frictional heat, which further increases the temperature of the grinding zone.
• Thirdly, low depth of cut can cause the grinding wheel to wear unevenly. Uneven wear can lead to surface roughness, as the wheel loses its sharpness and becomes less effective at removing material. Based on the above descriptions, in low depth of cut, temperature increase.

These notes should be presented that this steel have low thermal conductivity and absorb heat very slowly. Therefore, thermal gradients can easily cause cracking during heating or cooling. Therefore, the higher the machining temperature, the more cracks will occur during cooling. During machining with low depth of cut, the machining temperature increases more than high depth of cut. Therefore, a higher surface roughness is expected due to the creation of cracks. Therefore, extensive temperature changes during the machining process increase the surface roughness, which will be less in high flow rates of the coolant.

#### 4.2.2 Optimal conditions for surface quality

The best surface quality for the 1.2344, 1.2080, and 1.1191 steel alloys were obtained at cutting depths below 0.15 mm, a low cutting depth with the coolant flowing at 15 mL/s at 0 °C, and with the coolant flowing at 15 mL/s at 0 °C, respectively. Since the focus was on the effect of the proposed indirect cooling system on the workpiece surface roughness, we ignored the effect of lubrication, although the lubrication could expectedly improve the surface quality.

## 5 Conclusion

• This research introduces an indirect cooling grinding method (I.C.G.) as a means to attain workpieces with reduced surface roughness. To conduct an experimental investigation of the suggested method, the impact of heat transfer from the workpiece was examined by varying two parameters: coolant temperature and volumetric flow-rate. This analysis was performed across various cutting depths. Based on the findings, it is evident that the steel alloys possessing lower chromium content can be enhanced in terms of surface roughness through the implementation of the proposed I.C.G. Furthermore, it is noteworthy that additional beneficial outcomes can be achieved at lower chromium levels, surpassing the performance observed in the absence of any coolant. The investigation yielded the following conclusions:The best and worst surface roughness values were obtained with the 1.1191 and 1.2344 steel alloys at all coolant temperatures and volumetric flow-rates, respectively.
• The proposed method (I.C.G.) was more appropriate for the alloy with lower chromium content, in which case improved Ra was obtained upon reducing the coolant temperature. At higher chromium contents, however, excessive cooling of the workpiece was not of any help.
• Cooling some parts based on the chromium content can be convenient if it is controlled. Further cooling can improve Ra at all cutting depths if the chromium is too low.
• In the 1.1191 steel alloy, which is known to be free of chromium, the use of I.C.G. brought about 34 % improvement over the coolant-free condition. The chromium-containing steel alloys exhibited limited improvement, although the modifications were considerable.
• Using nanofluids instead of coolant, improving the flow path of the fluid from the bottom surface and modifying the heat exchanger can improve the performance of this method in the future works.

## Additional information

No additional information is available for this paper.

## CRediT authorship contribution statement

**Ali Heydari:** Writing – original draft, Visualization, Validation, Methodology, Formal analysis, Data curation, Conceptualization. **Masoud Pour:** Writing – original draft, Validation, Software, Investigation, Formal analysis, Data curation, Conceptualization. **Mohammad Reza Gharib:** Writing – review & editing, Writing – original draft, Validation, Supervision, Resources, Conceptualization.

## Declaration of competing interest

The authors declare that they have no known competing financial interests or personal relationships that could have appeared to influence the work reported in this paper.

## List of Abbreviation

**Abbreviation Definition**
I.C.G: Indirect Cooling Grinding
C.C.T: Continuous Cooling Transformation
C.S.: Cleaning System
GPNP: Graphene Nanoparticles
M.Q.L: Minimum Quantity Lubrication
NF.M.Q.L: Nanofluid Minimum Quantity Lubrication
POBCF: Plant-Oil-Based Cutting Fluid
VCRC: Vapor Compression Refrigeration Cycle
